# NOX4-reactive oxygen species axis: critical regulators of bone health and metabolism

**DOI:** 10.3389/fcell.2024.1432668

**Published:** 2024-08-12

**Authors:** Martina Dzubanova, Jacob M. Bond, Siobhan M. Craige, Michaela Tencerova

**Affiliations:** ^1^ Laboratory of Molecular Physiology of Bone, Institute of Physiology of the Czech Academy of Sciences, Prague, Czechia; ^2^ Faculty of Science, Charles University, Prague, Czechia; ^3^ Translational Biology, Medicine, and Health, Virginia Tech, Roanoke, VA, United States; ^4^ Human Nutrition, Foods and Exercise, Virginia Tech, Blacksburg, VA, United States

**Keywords:** bone marrow stromal cells, bone marrow adipose tissue, ROS, NADPH oxidase, bone fragility, obesity, senescence

## Abstract

Bone marrow stromal cells (BMSCs) play a significant role in bone metabolism as they can differentiate into osteoblasts, bone marrow adipocytes (BMAds), and chondrocytes. BMSCs chronically exposed to nutrient overload undergo adipogenic programming, resulting in bone marrow adipose tissue (BMAT) formation. BMAT is a fat depot transcriptionally, metabolically, and morphologically distinct from peripheral adipose depots. Reactive oxygen species (ROS) are elevated in obesity and serve as important signals directing BMSC fate. ROS produced by the NADPH oxidase (NOX) family of enzymes, such as NOX4, may be responsible for driving BMSC adipogenesis at the expense of osteogenic differentiation. The dual nature of ROS as both cellular signaling mediators and contributors to oxidative stress complicates their effects on bone metabolism. This review discusses the complex interplay between ROS and BMSC differentiation in the context of metabolic bone diseases.Special attention is paid to the role of NOX4-ROS in regulating cellular processes within the bone marrow microenvironment and potential target in metabolic bone diseases.

## Introduction

Bone marrow (BM) is the heterogenous and multicellular tissue located within the medullary cavity of bones. The BM plays an essential role in many physiological and pathological processes, including hematopoiesis, bone remodeling, and even cardiovascular and metabolic diseases (Benova and Tencerova, 2020). Although bone and BM are anatomically connected, they possess some specialized roles (Del Fattore et al., 2010). Bones provide skeletal support and organ protection, store calcium and phosphorus, and regulate various organ systems via the release of bone-derived hormones (“osteokines”), whereas the BM serves as a specialized niche that facilitates the generation of multiple crucial cell types, including red blood cells, white blood cells, and platelets (Del Fattore et al., 2010).

A significant stem cell population within the BM is hematopoietic stem cells (HSCs) (∼0.01%–0.1% of the total number of nuclear cells in BM aspirates) (Pang et al., 2011; Rossi et al., 2011). HSCs are the source of immune cell progenitors and bone resorbing cells (osteoclasts), which serve to negatively remodel bone. Beyond HSCs, multiple other cell types promote BM homeostasis, such as those providing nutrient supply (endothelial cells), innervations (nerve cells), and bone matrix formation (osteoblasts, osteocytes) and their progenitors, known as BM stromal cells (BMSCs) (Peci et al., 2022). BMSCs, though a rare population of cells within the BM (∼0.001%–0.01%) (Bianco et al., 2001), play a crucial role in the BM microenvironment as they can differentiate into osteoblasts, BM adipocytes (BMAds), and chondrocytes (cartrilage forming cells) (Dominici et al., 2006). The ability of BMSCs to differentiate into these different cell types is largely affected by physiological and pathophysiological conditions that result in the activation of different transcriptional programs and secreted factors (Tencerova and Kassem, 2016; Lecka-Czernik et al., 2017).

Within the BM, bone cells and adipocytes exhibit cell-to-cell contact and communication (Lanske and Rosen, 2017), to facilitate bone remodeling, hormonal regulation and nutrient exchange. BMAds collectively form BM adipose tissue (BMAT), a fat depot with unique molecular and physiological properties in comparison to peripheral adipose tissue (AT) (Suchacki and Cawthorn, 2018). During aging, up to 70% of red BM (replete with HSCs) undergoes conversion to yellow BM filled with BMAds (Kricun, 1985). This conversion occurs mainly in distal bones and does so centripetally (from peripheral skeleton to axial skeleton), possibly due to differences in temperature, vascularity, and oxygen tension in distal versus proximal bones (Kricun, 1985). In addition to aging (Farr et al., 2017), other metabolic diseases, such as obesity, diabetes and osteoporosis (Tencerova and Kassem, 2016; Suchacki and Cawthorn, 2018) affect BMSC properties and shift BMSC differentiation towards higher BMAd formation in the BM. Increased BMAT is often associated with reduced bone mineral density (BMD) and higher fracture risk, indicating a potential role of BMAT in the pathogenesis of osteoporosis (Shen et al., 2014; Beekman et al., 2023). Therefore, identifying the signals promoting BMAT accumulation in metabolic diseases could bring insight into the mechanisms affecting cell fate determination.

BMAT accumulation is accompanied with an increased production of reactive oxygen species (ROS), which contributes to a senescent BM microenvironment and increased bone fragility (Tencerova et al., 2019a). Traditionally, ROS were characterized simply as toxic by-products of aerobic metabolism that pathologically contributed to oxidative stress by damaging macromolecules such as lipids, proteins and nucleic acids (Beckman and Ames, 1998). As oxidative stress can accelerate various cellular processes including apoptosis and senescence (Jones, 2006), ROS production has been implicated in many disease processes. However, indiscriminate quenching of ROS can impair cell signaling as these molecules also can act as physiological signaling agents promoting health (Rhee et al., 2000; Finkel, 2011). Due to the two unpaired electrons in its outer orbital, oxygen (O_2_) is highly susceptible to the formation of ROS such as: O_2_^·−^, hydroxyl radical (HO^·^), H_2_O_2_, etc. (Sies, 2020). These ROS are characterized by different half-lives, charges and abilities to cross biological membranes. H_2_O_2_ is the most stable ROS as it has a significantly longer half-life and can cross biological membranes, enabling it to actively serve as a signaling molecule (Chen et al., 2008). For instance, H_2_O_2_ can directly react with cysteine residues of proteins involved in regulation of cell differentiation (e.g., Phosphatase and tensin homolog (PTEN), Akt2) (Vieceli Dalla Sega et al., 2017; Sies, 2020).

The family of nicotinamide adenine dinucleotide phosphate (NADPH) oxidase (NOX) enzymes is a major source of ROS production within the BM. It has been shown that NOX-ROS play an important role in cell proliferation, differentiation, homing, and senescence (Schroder, 2019). Notably, NOX members are found to be differentially expressed based on cell type, and their activity responds to specific extra- and intracellular signals through generation of ROS, e.g., superoxide (O_2_^·−^) or hydrogen peroxide (H_2_O_2_) (Brown and Griendling, 2009). Interestingly, NOX4 is unique among the NOX family members as it does not require agonist-stimulated activation to produce ROS (Martyn et al., 2006). A key structural property enabling NOX4 to facilitate essential cellular processes in its third extracystosolic loop (E loop). This loop allows NOX4 to produce H_2_O_2_ over O_2_^·−^ (Takac et al., 2011). Consequenly, NOX4-generated H_2_O_2_ may play a significant role in signal transduction (Finkel, 2011).

The role of NOX-ROS across different BM-resident cells in pathophysiological conditions is not well documented. Thus, this review provides an overview of the literature on elucidating the role of NOX-ROS in bone-fat metabolism. As NOX4 is important in BMSC differentiation (Atashi et al., 2015), we will highlight the potential role of NOX4 in the regulation of BMAT expansion and BMSC properties, which may significantly contribute to alterations in bone homeostasis.

### BMSCs and BMAT

The relationship between BMAT and metabolic diseases such as obesity and diabetes has recently attracted increased attention, even though BMAT was first described by anatomists in the late 19th century in histological sections of bone biopsies (Stockman and Greig, 1898). As opposed to the well-studied white, brown, beige and pink AT (Richard et al., 2000), the researchers have only recently begun to characterize the exact functions of BMAT. In the last decade, the BM adiposity literature has rapidly increased, leading to the recent understanding that BMAT acts as a unique fat depot that differs from peripheral AT not only anatomically but also developmentally, functionally, and metabolically (Hardouin et al., 2016; Suchacki and Cawthorn, 2018; Tencerova et al., 2018).

BMAT supplies energy to neighboring BM cells, such as osteoblasts and HSCs (Li et al., 2022; Alekos et al., 2023) during periods of increased energy demand, such as bone remodeling, haematopoiesis or cell proliferation (Shafat et al., 2017; Tabe et al., 2017; Tencerova et al., 2018). Beyond the traditional role of AT as an energy reserve, BMAT can contribute to bone loss through the release of pro-inflammatory and pro-resorptive cytokines and adipokines (e.g., receptor activator of nuclear factor kappa-Β ligand (RANKL), macrophage colony-stimulating factor (M-CSF), dipeptidyl peptidase (DPP4), lipocalin 2 (LCN2), tumor necrosis factor-alpha (TNF-α) and interleukin-6 (IL-6), which negatively regulate bone metabolism) (Herrmann, 2019; Tencerova et al., 2021). BMAT expansion results in compromised osteoblast differentiation, as BMAT originates from BMSCs (Tencerova and Kassem, 2016). This reciprocal relationship is further highlighted by the fact that molecular pathways promoting osteogenesis typically inhibit adipogenesis and *vice versa* (Li et al., 2013). This may be partially mediated by ROS-induced changes in redox-sensitive microRNAs that inhibit transcription factors such as Runt-related transcription factor 2 (RUNX2), impairing osteogenesis and augmenting adipogenesis in BMSCs through NF-κB signaling (Liao et al., 2013; Wang et al., 2015).

**FIGURE 1:**
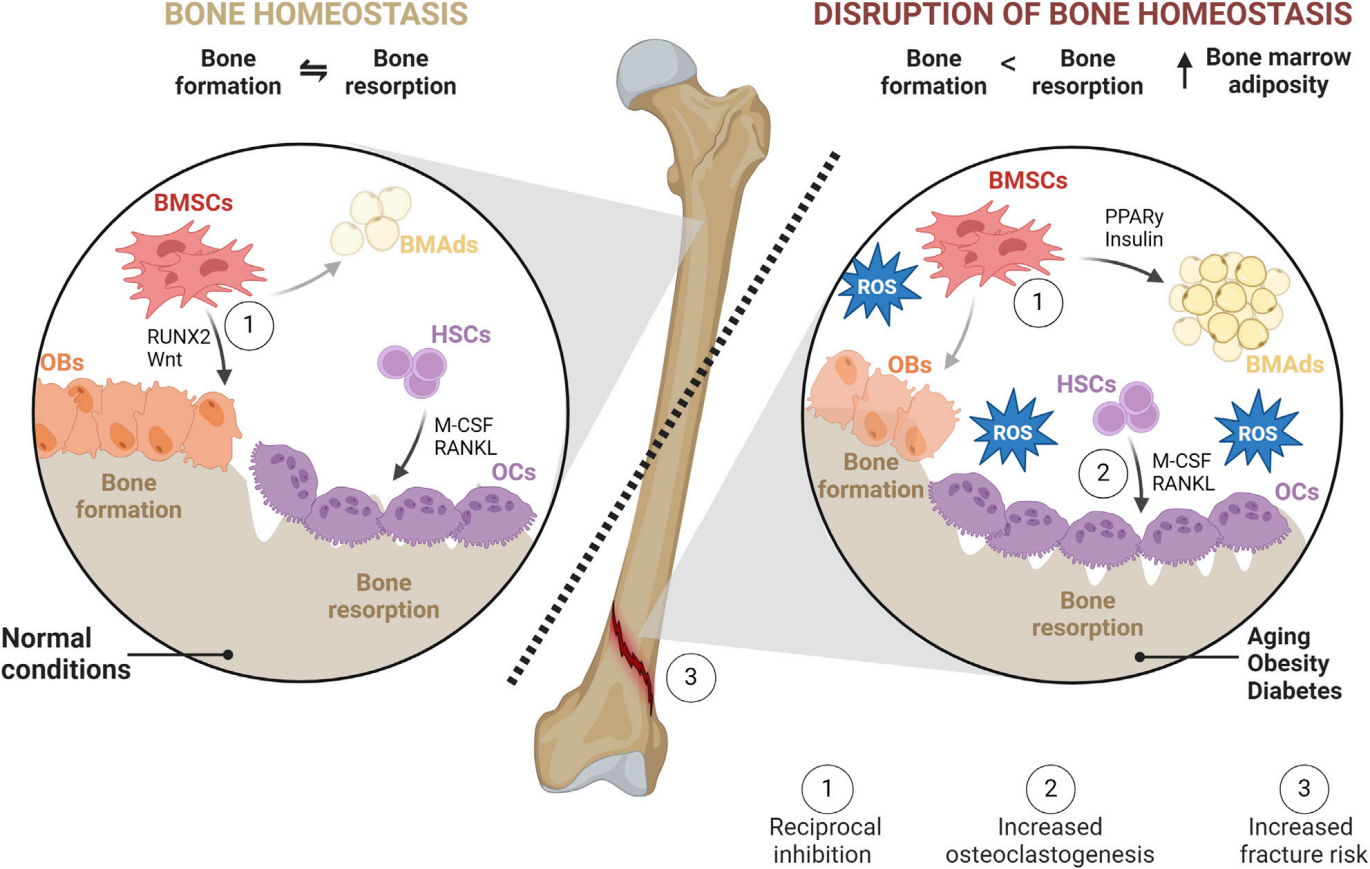
Bone homeostasis in normal and pathophysiological conditions. In physiological conditions, bone homeostasis represents a balance between bone formation (formed by OBs) and bone resorption (mediated by OCs). In homeostasis, BMSC differentiation favors OBs over BMAds. OCs, responsible for degrading bone, create lacunae that are subsequently filled with newly synthesized matrices by OBs. However, in pathophysiological conditions such as obesity, diabetes, or aging, this balance is disrupted. BMSC differentiation is shifted towards adipogenesis leading to the accumulation of BMAds within BM through increased PPARγ and insulin signaling. OB-mediated bone formation is diminished, while OC differentiation and activities, as well as subsequent bone resorption, are increased. This imbalance is facilitated directly or indirectly through increased production of RANKL and M-CSF, exacerbating the detrimental effects of oxidative stress on bone health and leading to a higher risk of fracture.

In both osteoporosis and obesity, there is commonly an imbalance in the regulation of osteoblastic and adipogenic BMSC differentiation (Sui et al., 2016). Several studies have shown a negative correlation between BMAT volume and BMD (Yeung et al., 2005; Li et al., 2014), which may underpin elevated risk of bone fractures (Woods et al., 2022; Guimaraes et al., 2023) (Figure 1). In obesity, BMAT resists the development of insulin resistance and inflammation, unlike what is observed in peripheral tissues (Tencerova et al., 2019a). However, continuous recruitment of BMSCs to BMAT as seen in obesity exceeds this protective potential and instead drives progenitor cell exhaustion, reduced osteoblastic recruitment, and ultimately decreased bone formation (Tencerova et al., 2018). Exposure of human BMSCs to sera isolated from overweight individuals increased adipocyte differentiation at the expense of osteogenic differentiation demonstrating that circulating factors are sufficient to skew the BMSC differentiation potential towards adipogenesis (Di Bernardo et al., 2014). In fact, the BM contains BMSCs that are uniquely primed for adipogenesis, which readily proliferate under obesogenic conditions and significantly contribute to BMAT expansion (Tencerova et al., 2019b). Increased ROS levels in obesity are associated with adipogenic BMSCs that demonstrate a shift from glycolysis towards higher oxidative phosphorylation, enhanced insulin signaling, glucose transport, lipid metabolism, and senescence (Guntur et al., 2018; Tencerova et al., 2019b). This hypermetabolic phenotype of BMSCs may represent a mechanism by which obesity contributes to bone fragility (Tencerova et al., 2019a). Thus, one can hypothesize that ROS might mediate the adverse effects of metabolic diseases on bone and BM microenvironment.

### ROS in the BM compartment

Emerging evidence suggests that elevated ROS in metabolic bone diseases negatively impact bone homeostasis (Table 1). ROS aid in mineralized matrix degradation and influence the behaviour of cells involved in this process (Agidigbi and Kim, 2019). Osteoclasts located on the bone interface generate O_2_^·−^and H_2_O_2_ which regulates their differentiation and development (Goettsch et al., 2013; Agidigbi and Kim, 2019). Furthermore, ROS released by neighboring cells stimulate osteoclast activity through ERK/NF-κB signaling and increased RANKL production (Ha et al., 2004; Lorenzo, 2017). These signals result in the inhibition of osteoblast lifespan (Deng et al., 2019), differentiation (Bai et al., 2004; Chen et al., 2018), and decreased alkaline phosphatase (ALP) activity (Luo et al., 2020). One of the key signaling pathways influenced by ROS is the Wingless/Int-1 (Wnt) signaling, important for BMSC fate, and homeostasis (Houschyar et al., 2018), which is diminished by excess ROS causing increased BMAd expansion (Atashi et al., 2015). ROS inhibition of Wnt occurs through the oxidation of key signaling molecules, such as β-catenin, which is crucial for Wnt signal transduction (Kajla et al., 2012; Staehlke et al., 2020).Another pathway affected by ROS is the PI3K/Akt pathway, which is vital for cell survival and proliferation (Liu et al., 2021). ROS can inhibit the activity of PTEN, a negative regulator of the PI3K/Akt pathway, leading to increased Akt signaling and altered cell survival and differentiation (Koundouros and Poulogiannis, 2018; Liu et al., 2021). ROS can modulate the MAPK/ERK pathway, which is involved in the regulation of cell growth and differentiation.

**TABLE 1:** Role of NOX-ROS in metsbolic bone diseases.

Metabolic bone disease	Role of NOX-ROS	References
Diabetes	NOX-ROS impair the bone vessels and bone mass, which leads to uncoupling of angiogenesis and osteogenesis and inhibition of bone formation	Wu et al. (2022)
Obesity	NOX-ROS impacts bone health by inducing oxidative stress in osteoblasts and altering bone remodeling	Halade et al. (2011), Rahman et al. (2018)
Osteoarthritis	NOX-ROS mediates chondrocyte apoptosis and matrix degradation, exacerbating cartilage destruction	Kim et al. (2015), van Dalen et al. (2018), Han et al. (2022)
Osteopetrosis	Dysregulated NOX-ROS levels impair osteoclast function, resulting in defective bone resorption and overly dense bones	Darden et al. (1996)
Osteoporosis	NOX-ROS contributes to bone resorption by osteoclasts, leading to decreased bone density and increased fracture risk	Joo et al. (2016), Schroder (2019), Sun et al. (2021)

The role of ROS in driving BMSC differentiation to BMAT is further confirmed through the use of antioxidants where osteogenic potential was enhanced while adipogenic potential was reduced in mouse and human BMSCs in response to antioxidants such as the flavanol quercetin (Wang et al., 2021), the fullerene-derivative fullerol (Liu et al., 2013), the polyphenol resveratrol (Ali et al., 2020), and the isoflavone formomentin (Gautam et al., 2017). The mechanisms by which ROS quenching reduces BMAT are still being elucidated but have been shown to involve interactions between canonical factors like RUNX2, osterix (OSX), RANKL and osteoprotegerin (OPG), which are crucial in bone remodeling (Wauquier et al., 2009; Ali et al., 2020).

**FIGURE 2:**
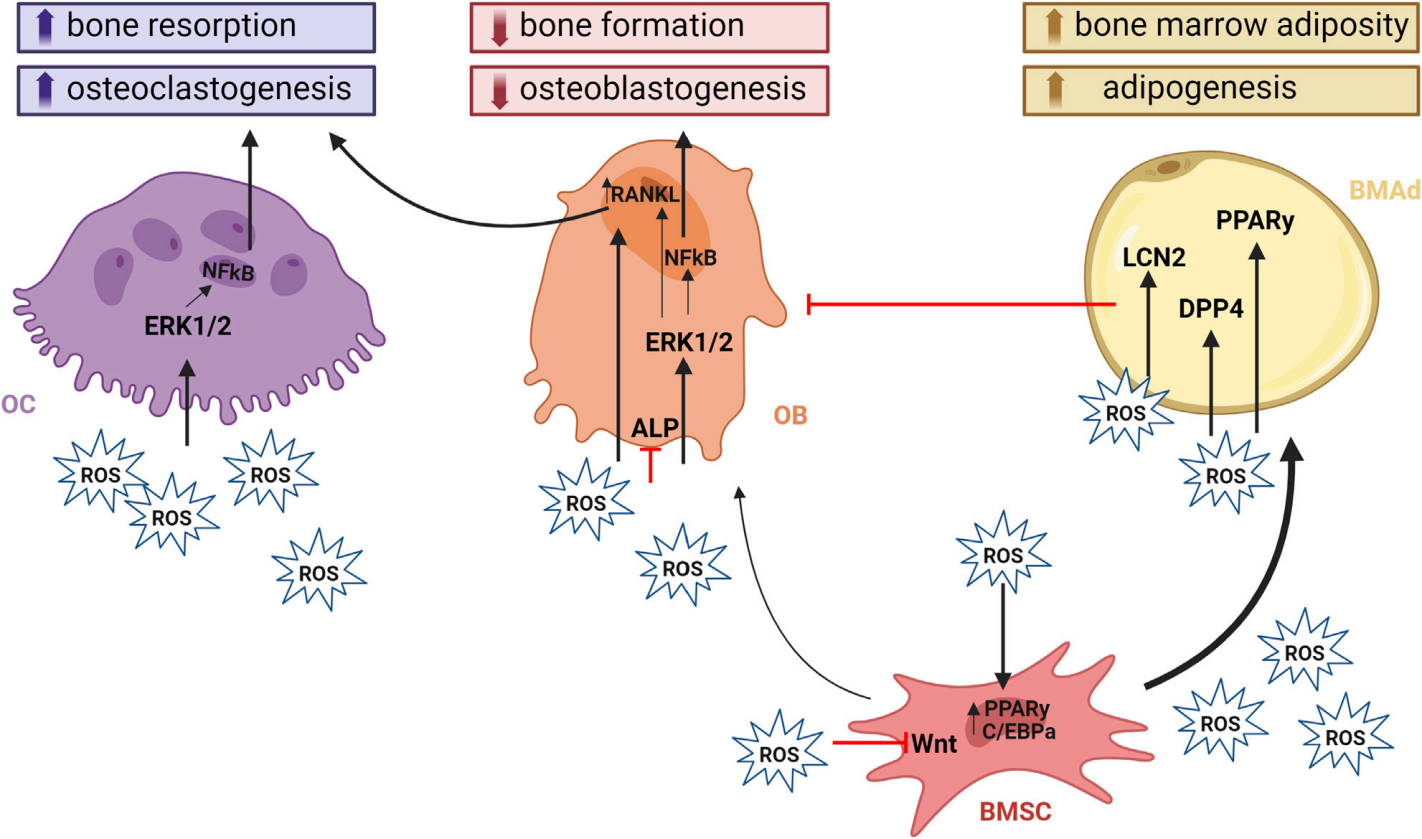
ROS and its effect on bone homeostasis. ROS play a crucial role in modulating signaling pathways within bone cells. They contribute to bone loss by impairing osteoblastogenesis and promoting osteoclastogenesis and adipogenesis. ROS induction of bone resorption occurs either directly by mediating OC function through activation of the mitogen-activated protein kinase (MAPK) signaling pathways such as JNK, p38, and ERK1/2. In addition, they have an important roles in signal transduction that activate cellular responses to many types of stresses, but also control the proliferation, differentiation, and survival of osteoclasts. Indirect activation of OCs is achieved through the upregulation of RANKL expression within OBs. PKA and CREB are central to the cAMP signaling pathway that regulates the production of RANKL. PKA activation leads to CREB phosphorylation, which binds to the RANKL promoter to enhance its transcription. This mechanism underscores the critical role of OBs in this process. On the other hand, osteoblastogenesis, together with bone formation is impaired through inhibiton of Wnt signaling and ALP activity, which are crucial for maintaining bone homeostasis. The activation of PPARγ promotes adipogenic differentiation of BMSCs at the expense of osteoblastogenesis. The presence of BMAds within the BM negatively affects the differentiaton of BMSCs towards OBs by releasing pro-inflammatory and pro- resorptive cytokines and adipokines. Created with BioRender.com

In summary, ROS play a pivotal role in bone by both promoting osteoclastogenesis and inhibiting osteoblast differentiation in favor of adipogenesis (Figure 2). While the role of ROS in bone remodeling and the impact on osteoclasts and osteoblasts are well-established, there is a critical need for comprehensive research to elucidate the specific ROS-producers and effects of ROS on BMSCs and, thus, BMAT. Unraveling the molecular mechanisms by which ROS are produced and may modulate BMSC fate decisions can provide valuable insights into the complex interplay between oxidative stress and BM homeostasis. This knowledge will not only contribute to a more nuanced understanding of bone physiology but may also unveil potential therapeutic targets for conditions characterized by altered bone homeostasis.

### NOX4-ROS signaling and its effect on BMSCs and BMAT function

The quenching of high levels of ROS via endogenous or exogenous antioxidants can prevent cell damage and attenuate BMSC apoptosis and loss of viability (Balogh et al., 2016). Yet, as described above, ROS are not only harmful by-products of cell metabolism but also participate in signal transduction and are required for cellular functions such as differentiation (Atashi et al., 2015). Therefore, it is critical to understand the sources and specify locations of ROS and their impacts on BMSC function. NOXs are considered a major source of ROS production within the BM. They are transmembrane proteins that transfer electrons across membranes to O_2_ using NADPH as an electron donor (Schroder, 2019). Among the NOX family members, research has highlighted the role of NOX2 and NOX4 in BM, demonstrating they contribute to bone loss, marrow adipogenesis, and osteoclastogenesis in mice (Atashi et al., 2015; Rahman et al., 2018; Sun et al., 2021), As osteoclastogenesis is intricately linked to BMSC function, the role of NOX4 in this process is significant. NOX4 is a critical source of ROS in mouse HSCs (Wang et al., 2010), human monocytes and human macrophages (Lee et al., 2010), controlling their function and differentiation (Yang et al., 2004). Notably, it has been reported that NOX4 limits bone mass by promoting osteoclastogenesis in an osteoporotic mouse model (Goettsch et al., 2013) and it is involved in the regulation of osteoprogenitors in bone development (Chen et al., 2022).

During differentiation, the major sources of ROS production include mitochondrial complexes I and III and NOX4 (Mahadev et al., 2001; Furukawa et al., 2004). Interestingly, the relationship between mitochondria and NOX4 seems to be bidirectional. In cancer cells, mitochondrial ATP produced through oxidative phosphorylation limits NOX4 activity by binding to a specific ATP-binding motif in the C-terminal tail of NOX4 (Shanmugasundaram et al., 2017), suggesting that NOX4 serves as an intracellular energy sensor. Indeed, NOX4 is required for mitochondrial biogenesis in the skeletal muscle following conditions of high energy demand like those in exercise (Specht et al., 2021). On the other hand, NOX4 has been shown to repress mitochondrial biogenesis and Complex I activity in fibroblasts (Bernard et al., 2017). As mitochondrial biogenesis increases during BMSC differentiation (Yan et al., 2021), understanding the relationship between NOX4, mitochondrial metabolism and mitochondrial biogenesis in BMSCs may be a fruitful avenue of research.

### NOX4-ROS and differential regulation on the peripheral AT and BM microenvironment in obesity

BMAT and peripheral AT are significantly different tissues (Liu et al., 2011; Miggitsch et al., 2019). However, examining NOX4 and ROS in peripheral AT may reveal crucial insights into NOX4 function and impact on overall metabolic health. In peripheral AT, NOX4 signaling pathways are primarily centered around adipogenesis and metabolic regulation (Den Hartigh et al., 2017). NOX4-ROS production stimulates the differentiation of preadipocytes to mature adipocytes (Schroder et al., 2009). This process involves the activation of adipogenic transcription factors which are essential for adipocyte maturation and lipid accumulation such as peroxisome proliferator-activated receptor gamma (PPARγ) and CCAAT enhancer-binding protein alpha (C/EBPα) (Schroder et al., 2009). In response to hypoxia, *Nox4* silencing in adipose-derived stem cells led to reduced proliferation and cell migration, along with decreased phosphorylation of platelet-derived growth factor receptor-β, AKT serine/threonine kinase 1 or Protein kinase B (AKT), and ERK1/2 (Kim et al., 2012). A common stressor to simulate obesogenic condition *in vitro* is high glucose. With obesity, NOX4 is upregulated in adipocytes (Den Hartigh et al., 2017). Peripheral preadipocytes differentiated from mice lacking adipocyte NOX4 are resistant to high glucose and palmitate-induced inflammation (Den Hartigh et al., 2017). This suggests that NOX4-ROS in AT participates in signaling cascades responsible for the early onset of insulin resistance and the inflammatory response in obesity (Den Hartigh et al., 2017). BMAds were shown to overproduce ROS mediated through the enhanced NOX4 expression, causing increased intracellular ROS levels and downregulating the endogenous antioxidant systems following high glucose treatment (Rharass and Lucas, 2019). Thus, BMAds are sensitive to both glucose and ROS levels, and these together influence their phenotype and functionality.

**FIGURE 3:**
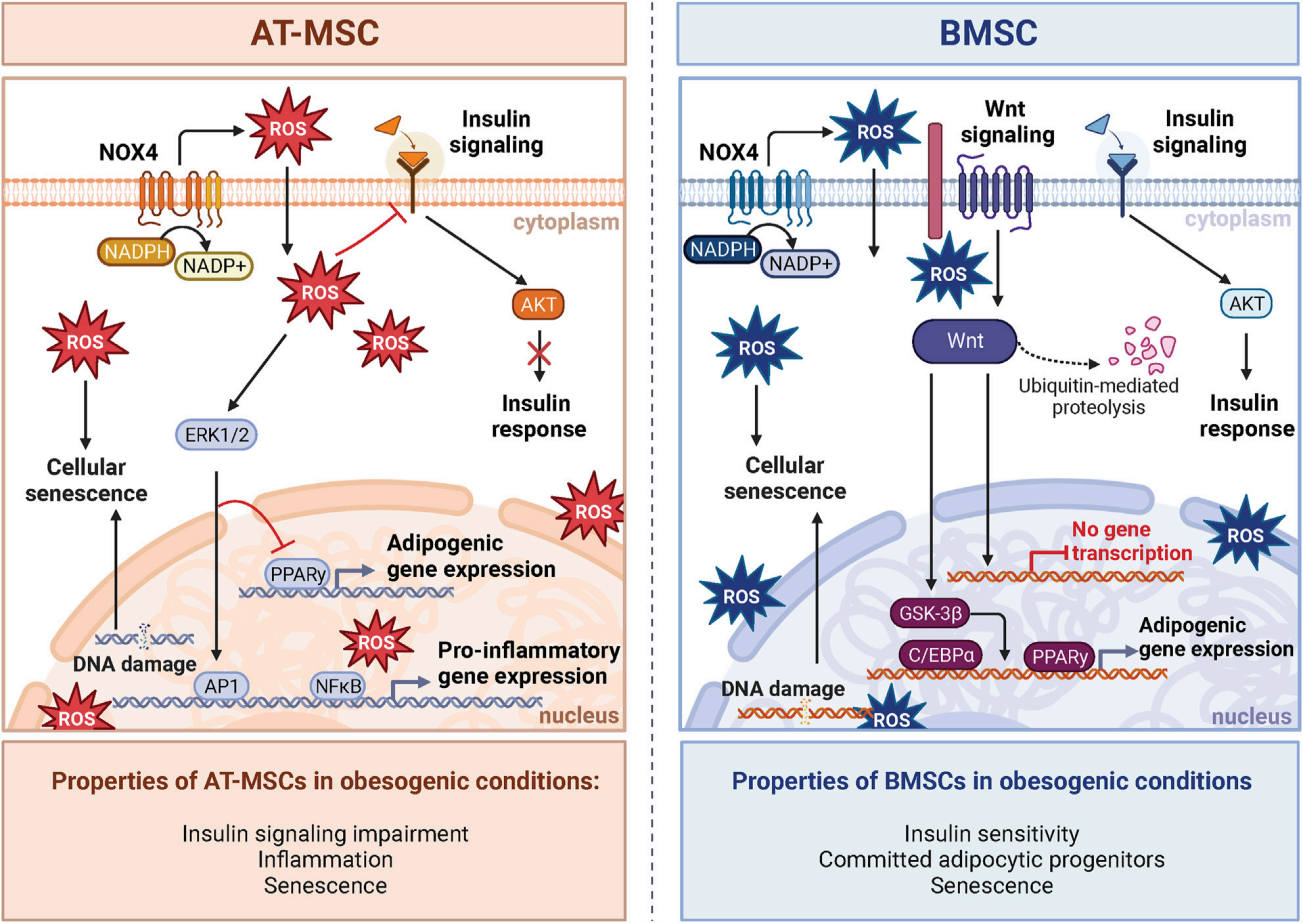
NOX4-ROS signaling in AT-MSCs and BMSCs. The impact of oxidative stress induced by obesogenic conditions on stem cells varies between adipose tissue-derived AT-MSCs and bone marrow-derived BMSCs. In AT-MSCs, NOX-ROS signaling pathways predominantly affect adipogenesis and metabolic regulation. In obesogenic conditions, the metabolism of AT-MSCs is characterized by compromised insulin response and increased inflammation caused by increased expression of NF-κB, leading to impaired adipogenesis and insulin resistance. On the other hand, BMSCs manifest a distinctive insulin response in obesogenic conditions defined in BM by the absence of inflammation, which leads to different insulin responsiveness and activation of AKT signaling compared to AT. Notably, unlike AT-MSCs, insulin signaling in BMSCs is enhanced in obesity. Obesogenic BMSCs exhibit a molecular phenotype shift towards committed adipocytic progenitors and inhibition of Wnt signaling, a critical factor for OB differentiation. Despite this, increased ROS contribute to an enhanced senescent phenotype in both cell types. Created with BioRender.com.

Comparing the transcriptome of BMAds and peripheral adipocytes demonstrates apparent differences between these two tissues (Suchacki et al., 2020). However, such a comparison may lend insights into the difference in response to ROS and adipogenic priming in obesogenic conditions discussed above. For instance, BMAds demonstrate increased early adipogenic gene expression, and lower late adipogenic genes compared to epididymal adipocytes (Liu et al., 2011). Furthermore, BMAds showed greater expression of pro-inflammatory genes (Liu et al., 2011) and displayed an elevated production of ROS (Miggitsch et al., 2019). These findings suggest that BMAds may be primed to receive maturation cues due to obesogenic stressors compared to peripheral adipocytes due to a comparably decreased antioxidant capacity. Therefore, NOX4-ROS may direct the maturation of BMSCs into BMAds within BM. Together, these data provide evidence that NOX4-ROS are important for activation, differentiation, and the response to metabolic stressors in the peripheral AT, which may also be relevant in the BM. Thus, these findings collectively indicate distinct responses of BMAds and peripheral adipocytes to metabolic stressors (Figure 3).

Differential responses between peripheral AT and BMAT may be due to NOX4 expression levels which alter downstream signaling. Transcriptomic profiling revealed that NOX4 is more highly expressed in AT compared to BM (data available from v23.0.proteinatlas.org; https://www.proteinatlas.org/ENSG00000086991-NOX4/tissue#rna_expression). This disparity may suggest regulation beyond the transcriptional level through varying activation and posttranslational modifications of proteins involved in downstream metabolic pathways between the two cell types (Forrester et al., 2018). Further investigation is needed to unravel the distinct role of NOX4 expression in producing the distinct phenotype between these two tissues and to define the unique role of NOX4 in determining the pathophysiology of BM and bone metabolism in obesity.

Obesity is a significant risk factor for insulin resistance, a maladaptive metabolic state characterized by impaired insulin-mediated glucose uptake, changes in insulin secretion and dyslipidemia (Czech, 2017). Importantly, increased NOX4-ROS in AT due to obesity promote the generation of dysregulated metabolism through increased production of adipokines such as plasminogen activator inhibitor 1 (PAI-1), IL-6, and monocyte chemotactic protein-1, and decreases the generation of the insulin-sensitizing factor, adiponectin (Furukawa et al., 2004). Systemic inflammation is another hallmark of obesity linked to poor bone health (Iantomasi et al., 2023). NOX4-ROS contributes to this low-grade inflammation in AT, where inflammation drives obesity-induced impairment of insulin signaling (Den Hartigh et al., 2017). However, mixed findings surround the idea that inflammation caused by obesity disrupts insulin signaling in BMAT. These observations underscore the complexity of how systemic metabolism impacts BMAT homeostasis and expansion (Pham et al., 2020). In obesity, the BM does not demonstrate a clear increase in the inflammatory response compared to the periphery (Tencerova et al., 2018). This suggests the existence of a barrier within the BM, likely due to a distinct microenvironment that significantly influences the the stress response of BM cells (Tencerova et al., 2018; Tencerova et al., 2019a). Indeed, recent animal and clinical studies (Tencerova et al., 2018; Pham et al., 2020) did not observe insulin resistance in obese BMSCs and BMAT, further supporting the hypothesis that significant metabolic and molecular differences exist in the BM compartment versus peripheral tissues. Another study reported that BMAT is capable of insulin-stimulated AKT S473 phosphorylation but lacks AKT T308 phosphorylation (Suchacki et al., 2020). These data suggest a distinct mechanism for lipogenesis in BMAT, possibly less dependent on insulin than in peripheral AT. Thus, these findings offer potential mechanistic insight into the differential responses between BMAds and peripheral adipocytes to metabolic stress (Figure 3). Recent studies using specific NOX4 inhibitors in osteoporotic animal models showed promising results in improvement of bone loss (Woods et al., 2022). Thus, targeting NOX4 in obesity-induced bone fragility may be an interesting target for potential treatment in patients with metabolic bone diseases. However, further studies are needed to better understand the underlying mechanism in the regulation of cellular metabolism and inflammatory responses in BMAT in the context of obesity. While NOX4 generates ROS in both peripheral AT and BMAT, the ultimate impact of NOX4-ROS in metabolic bone diseases appears to depend on the distinct depot and microenvironments within these tissues.

### Limitations of the current research studies

BMAT is heterogeneous: There are different types of BMAT (constitutive vs. regulated), present in different regions of the BM (Li et al., 2018) exhibiting unique properties and responses to ROS. The studies might not account for this heterogeneity, potentially oversimplifying the conclusions.

*In vivo* models are lacking: The versatility of *in vitro* models has promoted significant gains in our understanding of the impact of ROS on BMSC differentiation and other instrumental properties of BM cells. However, they also demonstrate the need for *in vivo* validation due to the widely recognized microenvironmental nuances, the phenotypic heterogeneity and multifaceted roles of BMSCs within the BM niche. Very little *in vivo* research is available using cell-type-specific genetic modifications or target-specific molecules to elucidate the role of ROS on BMAT in osteoporosis and metabolic diseases.

Sexual dimorphism requires further exploration: Research on females and female-derived cells underrepresent the current knowledge of ROS and BMAT. Due to known sexual dimorphisms in BM adiposity, redox homeostasis, and BMSC properties, further investigation is necessary to understand these differences and their implications (Malorni et al., 2007; Lecka-Czernik et al., 2017; Beekman et al., 2022).

Translation to human physiology: Findings from animal models and *in vitro* studies may not always translate directly to human physiology. Differences between species and the controlled experimental conditions can limit the applicability of the results to clinical settings.

## Conclusion and perspectives

**FIGURE 4:**
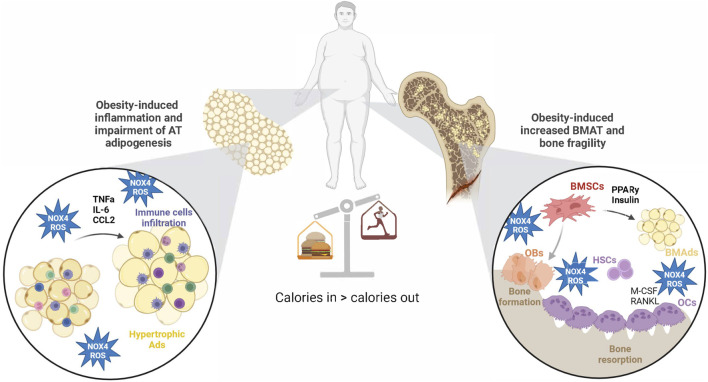
Obesity-induced changes in AT and BM microenvironment via NOX4-ROS production. Obesity increases NOX4-ROS production in AT, which affects adipogenesis and AT inflammation. On the other hand, NOX4-ROS in BM microenvironment accelerates BMSC adipogenesis at the expense of osteogenic differentiation. Those changes lead to impaired glucose metabolism and increased bone fragility in obesity. Created with BioRender.com.

Taken together, the recent discoveries provide a strong rationale for closer exploration of NOX4-ROS signaling in BMSCs and BMAT, as well as its unique functions when compared to peripheral AT. The BM is rich in progenitors sensitive to cues for adipogenesis in response to stressors such as metabolic diseases and aging, perhaps mediated by NOX4-ROS. Increased evidence in the literature suggests that NOX4-ROS could drive bone fragility in obesity by influencing BMSC senescence, proliferation, and adipocyte differentiation, ultimately promoting the expansion of BMAT (Figure 4). However, the exact signaling pathways and effects of NOX4 in BMAT are still being elucidated. They likely involve interactions with factors like RANKL and OPG, which are crucial in bone remodeling. More mechanistic and clinical studies investigating tissue-specific NOX4-ROS signaling may bring a better understanding of the role of NOX4 in the regulation of bone-fat metabolism and its potential use in the treatment of metabolic bone disease.

## Figures and Tables

**Table:** 

**AKT**	AKT serine/threonine kinase 1 or Protein kinase B
**ALP**	Alkaline phosphatase
**AP-1**	Activator protein 1
**AT**	Adipose tissue
**AT-MSCs**	Adipose tissue-derived mesenchymal stem cells
**BM**	Bone marrow
**BMAds**	Bone marrow adipocytes
**BMAT**	Bone marrow adipose tissue
**BMSCs**	Bone marrow stromal cells
**C/EBPα**	CCAAT enhancer-binding protein alpha
**CREB**	cAMP response element-binding protein
**DPP4**	Dipeptidyl peptidase
**ERK1/2**	Extracellular signal-regulated kinases 1 and 2
**GSK3β**	Glycogen synthase kinase-3 beta
**H_2_O_2_**	Hydrogen peroxide
**HO^·^**	Hydroxyl radical
**HSCs**	Hematopoietic stem cells
**IL-6**	Interleukine 6
**LCN2**	Lipocalin 2
**M-CSF**	Macrophage colony-stimulating factor
**NADPH**	Nicotinamide adenine dinucleotide phosphate
**NF-κB**	Nuclear factor kappa-light-chain-enhancer of activated B
**NOXs**	NADPH oxidases
**NOX2**	NADPH oxidase 2
**NOX4**	NADPH oxidase 4
**O_2_**	Molecular oxygen
**O_2_^·−^**	Superoxide
**OBs**	Osteoblasts
**OCs**	Osteoclasts
**OPG**	Osteoprotegerin
**PAI-1**	Plasminogen activator inhibitor 1
**PPARγ**	Peroxisome proliferator-activated receptor gamma
**RANKL**	Receptor activator of nuclear factor kappa-Β ligand
**RUNX2**	Runt-related transcription factor 2
**ROS**	Reactive oxygen species
**TNF-α**	Tumor necrosis factor-alpha
**Wnt**	Wingless/Int-1
